# Weekly platinum chemotherapy for recurrent ovarian cancer

**DOI:** 10.1038/sj.bjc.6600062

**Published:** 2002-01-07

**Authors:** A Clamp, G C Jayson

**Affiliations:** CRC Department of Medical Oncology, Christie Hospital NHS Trust, Wilmslow Road, Withington, Manchester M20 4BX, UK

## Abstract

*British Journal of Cancer* (2002) **86**, 2–4. DOI: 10.1038/sj/bjc/6600062 www.bjcancer.com

© 2002 The Cancer Research Campaign

## 

Although advanced ovarian carcinoma (FIGO stages III–IV) is sensitive to chemotherapy, with response rates of 70–80% following treatment with platinum/taxane combinations in first line regimens, the majority of patients relapse and their management at this stage is often unclear. A plethora of cytotoxic agents have been shown to have activity in this group in the last decade. In patients relapsing within 12 months of first-line chemotherapy, response rates to these agents however, are in the order of 20% with progression free intervals of 5–9 months only (Gore, 2001).

The most important predictive factor for response to chemotherapy is the progression-free interval, a phenomenon first described by Blackledge *et al* (1989). The response to phase II agents was correlated with time since last treatment. Patients whose disease progressed less than 6 months from their last chemotherapy had a response rate of less than 10% whilst those more than 18 months from treatment had a 94% chance of responding. Subsequent work (Markman *et al*, 1991), confirmed that this is a general predictor of chemosensitivity and that response to rechallenge with a platinum-based therapy is highly dependent on treatment-free interval with only 27% of patients 5–12 months from completion of cisplatin therapy responding compared with 59% of those whose disease progressed more than 24 months after initial treatment.

At present, current practice is to rechallenge patients relapsing more than 6–12 months after first-line chemotherapy with platinum-based regimens and to consider those relapsing sooner or who have truly refractory disease for newer agents, preferably within a clinical trial.

## WEEKLY CISPLATIN AND ORAL ETOPOSIDE

The article presented by Van der Burg *et al* (2002) in this issue, may come to challenge this view. The authors report impressive response rates and progression free survival in platinum-sensitive, resistant and refractory disease when treated with weekly cisplatin and prolonged daily oral etoposide. One hundred and seven patients were treated with an induction therapy of cisplatin 50 or 70 mg m^−2^ dissolved in 3% sodium chloride on days 1, 8, 15, 29, 36 and 43 together with oral etoposide 50 mg daily on days 1–15 and 29–43. Patients whose disease remained stable or responded continued on oral etoposide for a planned further 6–9 cycles at the same dose for 21 consecutive days in 28. All patients had bidimensionally measurable disease on cross-sectional imaging. However only the 98 patients completing induction therapy were evaluated for response rather than an intention-to-treat analysis being performed.

Of the 38 patients with platinum-sensitive disease, 92% responded to therapy with a median survival of 26 months, 91% of the patients with platinum-intermediate disease (a platinum free interval of 4–12 months) responded with a median survival of 16 months. Importantly 46% of 28 patients with platinum-refractory disease (platinum free interval of less than 4 months) responded although the median duration of this was only 5 months with median survival being 13 months. Myelotoxicity during cisplatin administration was cumulative with grade III/IV leucopenia seen in 58% of patients receiving 70 mg m^−2^ and grade III/IV thrombocytopenia in 40%. However, only one case of neutropenic fever was documented and only 10% of patients needed more than a 1 week delay in treatment. Reported non-haematological toxicity was in general mild with nephrotoxicity limited to grade I/II in 4% of patients whilst grade II sensory neuropathy was only seen in 7%. Prolongation of etoposide monotherapy was associated with grade IV neutropenia in only 2% of cycles.

## PREVIOUS STUDIES

Several previous studies have demonstrated response rates of 0–27% in patients with platinum-resistant disease that were treated with oral etoposide monotherapy (Markman *et al*, 1992; Marzola *et al*, 1993; De Wit *et al*, 1994; Hoskins and Swenerton, 1994; Seymour *et al*, 1994; Kavanagh *et al*, 1995; Kuhn *et al*, 1996; Rose *et al*, 1998). The largest reported a response rate of 27% in 41 patients who had recurrent disease within 6 months of completing platinum-based chemotherapy although median survival was only 10.8 months (Rose *et al*, 1998).

Previous data for weekly cisplatin salvage therapy is sparse. Two early phase II studies showed response rates of 62.5 and 70% in eight and 10 patients respectively for its use as third line therapy (Piver *et al*, 1980; Scotto and Sbiroli, 1991). One larger study of 72 patients whose disease had previously responded to cisplatin reported an overall response rate of 70% to weekly cisplatin at 1 mg kg^−1^ for 9 weeks in combination with either intravenous etoposide, epirubicin or carboplatin. Of note, 20 of 33 patients whose disease had relapsed within 18 months of initial diagnosis responded (Bolis *et al*, 1994). Evidence for the use of dose-dense cisplatin as first-line chemotherapy is disappointing. Whilst Levin *et al* (1993) in their meta-analysis of dose intensity in ovarian cancer chemotherapy demonstrated a statistically significant correlation between response rate and cisplatin dose intensity, the greatest impact is at doses below 25 mg m^−2^ week^−1^ and two large studies comparing nine doses of weekly cisplatin 50 mg m^−2^ to either conventional 3-weekly cisplatin at 75 mg m^−2^ (Colombo *et al*, 1993) or combination cisplatin and cyclophosphamide (Bolis *et al*, 1997) showed equivalence only. One study of 101 patients however revealed a trend towards increased survival at 5 years in favour of weekly compared to 3-weekly cisplatin (Cocconi *et al*, 1999).

Importantly, Meyer *et al* (2001) present a retrospective analysis of 42 patients with relapsed ovarian cancer treated with a similar weekly regimen. Cisplatin (60 mg m^−2^) was administered on days 1, 8, 15, 29, 36 and 43 and 50 mg oral etoposide was given on days 1–14 and 29–43 although this was only continued for a maximum of two cycles after induction therapy in patients whose disease responded. Response rate was assessed by CA-125 criteria only and 46% of patients whose disease had relapsed within 6 months of platinum-based chemotherapy responded to this weekly regimen. However, only 43% of patients with a platinum-free interval of more than 6 months responded. The median survival was 6.3 and 6.9 months respectively in these groups and the toxicity was similar to that reported by Van der Burg *et al* (2002).

Whilst both Van der Burg *et al* (2002) and Meyer *et al* (2001) report impressive response rates in platinum resistant disease with weekly cisplatin and prolonged oral etoposide, how do we explain the large discrepancies in overall survival and response rates in platinum-sensitive patients in these studies? As well as the definitions of platinum-sensitive disease being different (platinum-free intervals of 12 and 6 months respectively), in the study by Meyer *et al* (2001) platinum-sensitive patients had received more prior chemotherapy (2.63 courses compared to 1.13) and had a shorter platinum free interval (10.5 months *vs* 25 months). Their patients were also treated outside of a clinical trial and so may be more representative of the diverse spectrum of patients seen in general clinical practice – however Meyer *et al* (2001) do not comment on tumour bulk or performance status which are prognostic factors that may also explain this finding (Eisenhauer *et al*, 1997; Gore, 2001).

## WEEKLY CHEMOTHERAPY: TOXICITY AND EFFICACY

Whilst the combination of the two approaches presented here demonstrates markedly better response rates than that seen with monotherapy in relapsed ovarian cancer, it is different from other combination treatments in that toxicity appears to be less marked. For instance, the combination of mitoxantrone and paclitaxel has been recently reported to have a response rate of 69% in a predominantly platinum-refractory population of 33 patients. Grade III/IV neutropenia was however seen in 64% of cycles (compared with 16% of cisplatin doses as reported by Van der Burg *et al* (2002)) and G-CSF support was required in 49% (Janat *et al*, 2000).

As our principal aim in treating relapsed ovarian cancer is palliative, careful attention should therefore be paid to toxicity when designing combination strategies. Administering chemotherapy weekly to modify the side-effect profile without decreasing efficacy is a well-established policy in other solid malignancies. In breast cancer, the administration of taxanes weekly in metastatic disease decreases markedly the incidence of significant myelosuppression and grade III/IV toxicities overall (Perez *et al*, 1999; Burstein *et al*, 2000). This approach has also been adopted in relapsed advanced ovarian cancer with 3-weekly (200 mg m^−2^) and weekly paclitaxel (67 mg m^−2^) being demonstrated to be equally efficacious in paclitaxel-naïve patients with markedly fewer toxicities apart from nail changes and lethargy in the weekly arm (Andersson *et al*, 2000). There is also preliminary evidence from two studies to suggest that patients refractory to both platinum and 3-weekly paclitaxel may respond to weekly administration (Belinson *et al*, 2000; Kaern *et al*, 2001). Whether this is due to more prolonged cancer cell exposure to the taxane or the emergence of other mechanisms of action such as an anti-angiogenic effect is unclear.

One concern persists with the use of prolonged oral etoposide, in particular in combination with a platinum. It is well established that topoisomerase II inhibitors are leukaemogenic by increasing the risk of balanced chromosomal translocations. Etoposide is classically associated with *de novo* onset acute myeloid leukaemia characterized by a rearrangement involving the MLL gene on Chromosome 11q23 that has a latency of 2–3 years (Felix and Megonigal, 2001). Whilst only one case was reported in the GOG study discussed above (Rose *et al*, 1998) and none in this, it has been estimated that the cumulative risk with prolonged oral exposure may be as high as 4% (Pui and Relling, 2000). Although this consideration may not be prominent in the treatment of recurrent ovarian cancer attempts to evaluate this regimen in the first line treatment of the disease would be limited by concern over long term myelotoxicity and therefore the evaluation of weekly cisplatin with, for example, a taxane would be of great interest. Notably, two preliminary reports have already demonstrated the feasibility and promise of this approach in both platinum-sensitive and -resistant disease with acceptable toxicity (Bolanos *et al*, 2001; Katsumata *et al*, 2001).

## CONCLUSIONS AND FUTURE STUDIES

Two studies have reported striking response rates in women with platinum-refractory disease who were treated with weekly cisplatin and etoposide. Van der Burg *et al* (2002) suggest that all patients with recurrent disease should therefore be considered for rechallenge with weekly cisplatin. To what extent do their data support this thesis? The correlation between response to chemotherapy and patient survival is variable and with regard to weekly cisplatin and etoposide this will only be unravelled in the context of a randomized phase III trial. Such a study should also take the opportunity to collate detailed quality of life data given that recurrent ovarian cancer is generally incurable. It will also evaluate the importance of prior taxane exposure, an issue not addressed in this study as these agents only entered routine first line use in the last 5 years.

If the response rates shown in this study are confirmed, it will be important to re-evaluate the use of chemotherapy in relapsed ovarian cancer associated with bowel obstruction. This is a common and difficult problem and its course proves extremely distressing to both patients and their family. Previous studies have failed to show a benefit with chemotherapy but the development of a regimen with a high response rate, in particular if this is associated with a short time to response, would be a vital addition to our current management strategy.
